# Immunobiology of Steroid-Unresponsive Severe Asthma

**DOI:** 10.3389/falgy.2021.718267

**Published:** 2021-08-27

**Authors:** Courtney Lynn Marshall, Kosovare Hasani, Neeloffer Mookherjee

**Affiliations:** ^1^Department of Internal Medicine, Manitoba Center of Proteomics and Systems Biology, University of Manitoba, Winnipeg, MB, Canada; ^2^Department of Immunology, Rady Faculty of Health Sciences, University of Manitoba, Winnipeg, MB, Canada

**Keywords:** airway inflammation, asthma, steroid-resistance, glucocorticoids, immunobiology

## Abstract

Asthma is a heterogeneous respiratory disease characterized by airflow obstruction, bronchial hyperresponsiveness and airway inflammation. Approximately 10% of asthma patients suffer from uncontrolled severe asthma (SA). A major difference between patients with SA from those with mild-to-moderate asthma is the resistance to common glucocorticoid treatments. Thus, steroid-unresponsive uncontrolled asthma is a hallmark of SA. An impediment in the development of new therapies for SA is a limited understanding of the range of immune responses and molecular networks that can contribute to the disease process. Typically SA is thought to be characterized by a Th2-low and Th17-high immunophenotype, accompanied by neutrophilic airway inflammation. However, Th2-mediated eosinophilic inflammation, as well as mixed Th1/Th17-mediated inflammation, is also described in SA. Thus, existing studies indicate that the immunophenotype of SA is diverse. This review attempts to summarize the interplay of different immune mediators and related mechanisms that are associated with airway inflammation and the immunobiology of SA.

## Introduction

Asthma is a complex, respiratory disease characterized by airway inflammation and bronchoconstriction, which make it difficult to breathe. Asthma affects ~300 million people worldwide (1). There are heterogeneous clinical symptoms with varying degrees of response to therapy in asthma. Inhaled corticosteroid (ICS) is a common therapy for asthma, to which controllers such as a long-acting β2 agonist (LABA) are added if required, and if these fail oral corticosteroids are also added (2, 3). Approximately 10% of asthma patients do not respond to available steroid treatments (2). In 2014, a task force of ERS/ATS defined severe asthma (SA) as “asthma which requires treatment with high dose inhaled corticosteroids (ICS) plus a second controller (and/or systemic corticosteroids) to prevent it from becoming ‘uncontrolled,' or which remains ‘uncontrolled' despite this therapy” (2). Although patients with SA make up a small proportion of asthma patients, this subgroup accounts for more than 50% of direct and indirect healthcare costs associated with asthma (1). Research in the last three decades has shown that there are multiple phenotypes or subgroups in SA, with differences in clinical symptoms and molecular profiles. There is also a prominent sex-related disparity, as SA disproportionally affects adult females compared to males. Some studies demonstrate that almost 2/3rd of severe asthmatics are females (4). Resistance to corticosteroids can be attributed to a variety of components, from genetic variability to various molecular factors such as defective glucocorticoid receptor (GR) function with increased expression of the non-responsive isoform of GRβ, different transcription factor and signaling pathways, as well as specific cytokine-mediated downstream responses (5–7). The repertoire of immunological response that contributes to the pathophysiology in SA is diverse, and not well characterized. In this review, we attempt to summarize the immunobiology of airway inflammation and related molecular mechanisms that contribute to SA.

### Inflammatory Phenotypes Associated With Severe Asthma

Asthma was traditionally classified as a disease with an increase in predominantly T-helper (Th) 2 cells and elevated abundance of Th2-related cytokines namely IL-4, IL-5 and IL-13. Persistence of elevated levels of these typical Th2 cytokines does not necessarily correlate with disease severity in SA (8). Current understanding indicates that the inflammatory phenotypes in SA can be Th2-low as well as Th2-high (9–11). Inflammatory profile that is Th2-low with Th17-high responses, accompanied by dominant neutrophilia, is primarily defined for SA (11–14). However, some SA patients also demonstrate a Th2-high inflammation with persistent airway eosinophilia (6). Emerging studies have started to unravel the heterogeneity based on the abundance of different types of leukocytes and cytokines in the lungs and sputum of SA patients. A variety of immunophenotypes which includes Th2-low/Th17-high, Th2-high or mixed Th1/Th17 inflammatory profiles have been demonstrated in SA. There is an increase in airway granulocytes, neutrophils and eosinophils (15), with significantly higher neutrophil accumulation in the airways and sputum in patients with SA (6, 16–18). The percentage of neutrophils in the sputum of patients with SA is ≥40% higher than those with mild to moderate asthma (19). Importantly, the extent of neutrophilic inflammation has been shown to be positively associated with the severity of the disease and steroid-unresponsiveness in SA (17, 19, 20). Th17- and IL-17-mediated cellular mechanisms are primary drivers of neutrophil recruitment in the airways of patients with SA (6, 20). Interestingly, some patients with SA also show airway subepithelial cells expressing significantly higher IFNγ and IL-8, and lower IL-4 (Th2-low), compared to those with moderate and treatment-responsive disease, suggesting the occurrence of a Th1-skewed inflammatory disease phenotype in SA (16). Unfortunately, there are no clinically accepted biomarkers for Th2-low SA characterized with neutrophilic inflammation. Although neutrophilia is predominantly associated with SA, clinical trials with a selective CXCR2 antagonist (developed by AstraZeneca) significantly reduced neutrophil infiltrate but did not show any benefits in clinical outcomes of SA (21), further indicating that diverse leukocyte-mediated mechanisms can contribute to the disease phenotype.

SA patients with Th2-high inflammation show persistent airway eosinophilia, also termed as late-onset eosinophilic asthma (6). The eosinophil positive subtype of SA is associated with an increase in CD3+ CD4+ CD8+ T-cells, mast cells and macrophages (15). It remains unclear how this subtype of asthma patients with a Th2-high inflammatory phenotype are resistant to steroid treatments. Recent studies suggest that IL-33 produced by airway epithelial cells activate innate lymphoid cells 2 (ILC2). These IL-33-activated ILC2s produce Th2-cytokines IL-4, IL-5, and IL-13, leading to eosinophilic asthma which is steroid-resistant (6, 22). The molecular mechanisms that underpin steroid-resistance in an eosinophilic airway inflammation induced by the IL-33-ILC2 axis are not entirely defined. Nevertheless, IL-33 is significantly elevated in the airways of patients with steroid-unresponsive asthma (23–25), is known to mediate glucocorticoid resistance (23, 26), and being examined as a biomarker for SA (described below). Although there are several biomarkers described for Th2-high eosinophilic inflammation such as FeNO, blood total eosinophil count and eosinophil-derived neurotoxin, longitudinal cohort studies have shown that none of these biomarkers can sufficiently differentiate the phenotypes/endotypes in SA (20). Due to the diversity of inflammatory phenotypes associated with SA, it is important to unravel the immunobiology of this disease. Here, we further summarize some of the mechanisms associated with neutrophilic airway inflammation in SA.

### Promotion of Airway Inflammation by Neutrophils in Severe Asthma

**Figure 1:**
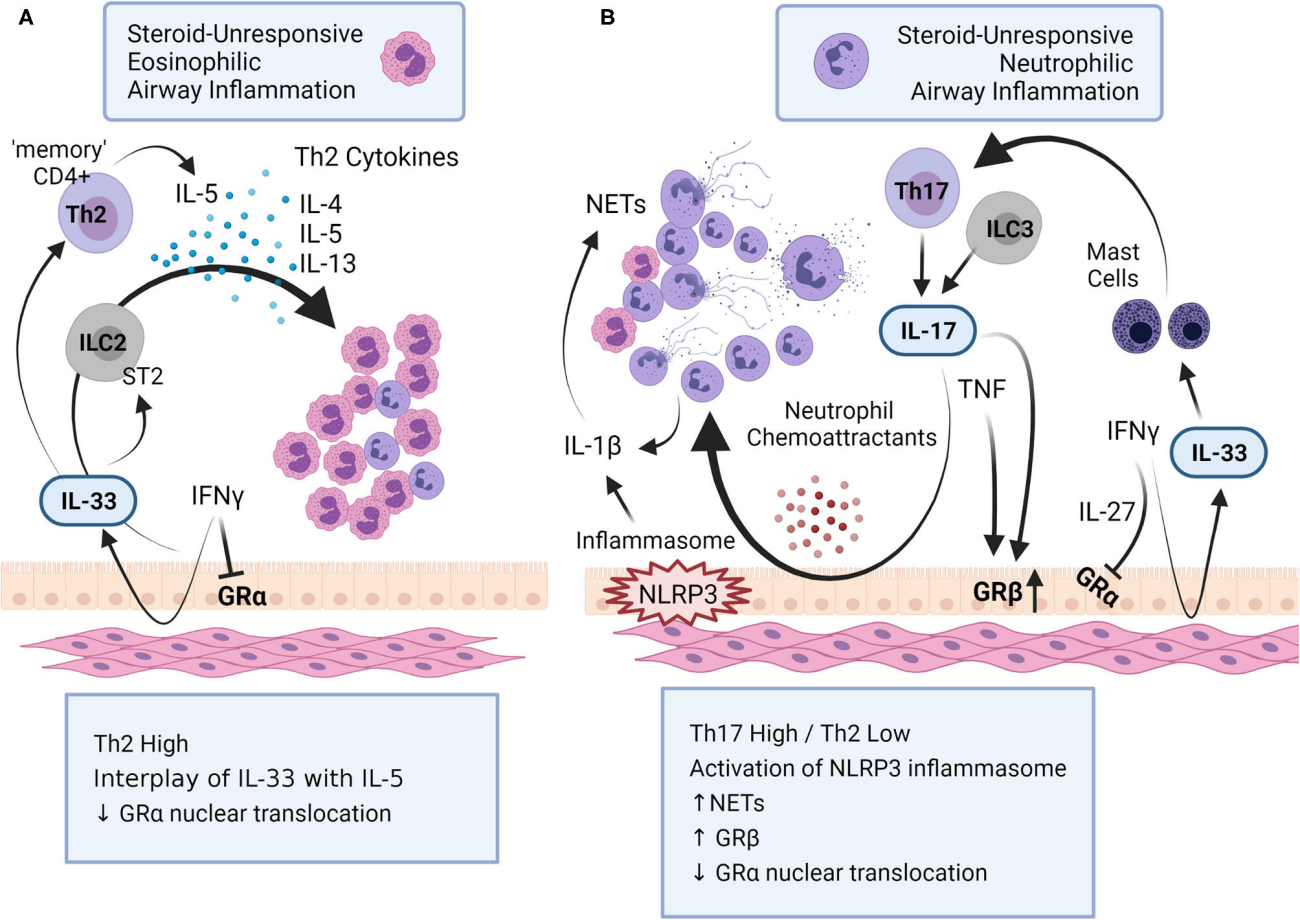
Mechanisms of steroid-unresponsive airway inflammation. Steroid-unresponsive severe asthma (SA) includes both **(A)** eosinophilic and **(B)** neutrophilic airway inflammatory phenotypes. **(A)** Eosinophilic airway inflammation in SA is characterized by a Th2-high inflammatory phenotype with increased levels of Th2-cytokines such as IL-4, IL-5 and IL-13 that promote increased accumulation of eosinophils in the lungs. The alarmin IL-33 interacts with innate lymphoid cells (ILC) 2 to facilitate the production of Th-2 cytokines. In addition, chronic exposure to IL-33 results in “memory” CD4+ Th2 cells that preferentially produce IL-5. **(B)** Neutrophilic airway inflammation in SA is typically characterized with a Th17-high and Th2-low inflammatory phenotype, with increased accumulation of neutrophils in the lungs. Different cytokines, namely IL-17, IFNγ, TNF and IL-1β, as well as the activation of inflammasome NLRP3, play a critical role in promoting neutrophilic airway inflammation in SA. Cytokines associated with both these immunophenotypes in SA dysregulates glucocorticoid receptor (GR) function; IFNγ and IL-27 suppress the nuclear translocation of GRα, the GR isoform that regulates glucocorticoid-mediated anti-inflammatory gene expression. IL-17 and TNF increases the expression of GRβ, isoform that attenuates GRα. This figure summarizes some of the key mechanisms related to airway inflammation in SA (created with BioRender.com).

Neutrophils promote airway inflammation in SA by several mechanisms (Figure 1). Activated neutrophils promote dysregulation of lipid mediators of inflammation such as the ceramide/sphingosine-1-phosphate pathway, which results in further recruitment of neutrophils and eosinophils, thus amplifying airway inflammation in SA (27). Neutrophils release pro-inflammatory cytokines such as TNF and IL-1β that have been associated with SA (12, 28). Neutrophilic airway inflammation also correlates with increased expression of the NLRP3 inflammasome and IL-1β in SA patients (28, 29). A study by Kim et al showed that steroid-unresponsive neutrophilic airway inflammation is promoted by NLRP3 inflammasome, mediated primarily through the activation of caspase 1 and subsequent enhancement of IL-1β (29). In the study by Kim et al., inhibition of caspase 1 and suppression of IL-1β alleviated steroid-unresponsive neutrophilic airway inflammation in an animal model. This is corroborated by another study which showed that sputum of patients with SA have high levels of Neutrophil Extracellular Traps (NETs)-derived extracellular DNA, with concurrent activation of inflammasome marker caspase 1 in the airways (30). NETs are essentially a lattice of chromatin fibers released from activated neutrophils which contain DNA, histones, granule-derived antimicrobial peptides and enzymes such as myeloperoxidase and neutrophil elastase. Several studies have demonstrated NETs-mediated mechanisms in the enhancement of airway inflammation and subsequent airway epithelial cell damage in SA (6, 30, 31). NETs can induce pro-inflammatory cytokines by stimulating macrophages, which further promotes neutrophil infiltration, thus generating a feedback loop for amplifying airway tissue damage in neutrophilic asthma (32). Interestingly, a NETs-independent mechanism mediated by enucleated neutrophil cytoplasts via the activation of dendritic cells, and driven by IL-17, was demonstrated in an animal model of neutrophilic airway inflammation (33). However, neutrophils, NETs and neutrophil cytoplasts, have all been shown to correlate with IL-17 levels in the airways, and are increased in the lungs of SA patients (6, 33). It thus remains unclear if NETs and/or neutrophil cytoplasts are essential for the development and persistence of SA characterized with neutrophilic airway inflammation.

### Neutrophil Proteins and Airway Remodeling in Severe Asthma

Neutrophils are a major source of the proteolytic enzyme matrix metalloproteinase (MMP) 9, also known as gelatinase B, which can degrade extracellular matrix. MMP9 degrades collagen type IV of the vascular basement membrane, and promotes biological processes that contribute to the decline of lung function connected to airway hyperresponsiveness (AHR) and airway remodeling (34–36). Neutrophil-derived MMP9 is increased in the bronchoalveolar lavage fluid (BALF) and sputum of patients with SA (37, 38). The amount of MMP9 in BALF directly correlates with the disease severity and decline in lung function in asthma (39, 40). A recent study using a treatment with omalizumab, an anti-IgE monoclonal antibody, demonstrated that a decrease in BALF levels of MMP9 correlates with a lower asthma exacerbation rate in SA patients (41). Interestingly, although the decrease of MMP9 abundance in BALF correlates with reduction in reticular basement membrane thickness, it does not alter collagen or fibronectin accumulation in SA patients (41). Proteomics and transcriptomics studies have demonstrated that the neutrophil-derived protein neutrophil elastase (NE) is upregulated in the sputum of SA patients (42). NE is known to inhibit tissue inhibitors of metalloproteinases 1 (TIMP1), which is an inhibitor of MMP9, thus facilitating an increase in MMP9 and subsequent decline in lung function (43). Aligned with this, a dysregulated balance of the MMP9/TIMP1 ratio in sputum has been shown to associate with airway remodeling and asthma exacerbation (44, 45). Another study showed that imbalance in MMP9/TIMP1 ratio in serum associates with reduced responsiveness to steroids (46). It has been shown that ICS is ineffective in reducing MMP9 levels or controlling MMP9 activity, thus substantiating a functional role of MMP9 in steroid-unresponsiveness in SA (47, 48). Therefore, unraveling mechanisms that underpin MMP9 activity and subsequent airway remodeling may provide insight for the development of new intervention strategies to mitigate steroid-unresponsiveness in SA. A caveat to consider is that although TIMP1 and MMP9 enzyme activities have been associated with decline in lung function, the ratio of MMP9/TIMP1 does not necessarily correlate with the disease severity in asthma (49). As MMP9 primarily promotes pulmonary fibrosis linked to airway inflammation (35, 36), it may well be that MMP9 may not be a critical mediator of AHR wherein the process is independent of airway inflammation. Thus, targeting MMP9 or its downstream activity may not be fully effective for the control of airway remodeling in SA. This highlights the need for a better understanding of the molecular mechanisms that underpin the biological process of airway remodeling, and that it should be considered independently from airway inflammation. This is consistent with recent studies suggesting that airway remodeling and AHR may be governed by inflammation-independent biological processes (50, 51).

### Cytokines in Steroid-Unresponsive Airway Inflammation

The cytokine networks that facilitate and amplify the disease in SA have not yet been completely defined. Cytokines enhanced in the BALF obtained from children and adults with SA are often associated with neutrophilic inflammation, with primarily either a Th17-high or a mixed Th17/Th2/Th1 immunophenotype (17, 52, 53). It is critical to unravel the role of specific cytokines and interacting protein partners that promote airway inflammation, AHR and the disease pathophysiology in SA, to gain a better understanding of immunomodulatory pathways that may be targeted for new interventions. Cytokines that are dominant in Th2-high inflammation such as IL-4, IL-5 and IL-13, are typically associated with eosinophil-skewed responses that are steroid-sensitive (54). However, the interplay of IL-5 with IL-33 contributes to late-onset eosinophilic asthma that is unresponsive to steroids (6). Furthermore, a recent study showed that SA patients with bacterial dysbiosis in peripheral airways have high levels of IL-13 in BALF along with neutrophilia (14). It is likely that the cytokine profile in SA is related to subclinical infections in the lungs (52). However, this is disputed by a study demonstrating that the BALF cytokine profile in neutrophilic SA is independent of respiratory pathogens (53). Thus, the association of airway cytokine profile and the microbiome in SA is not well understood, and needs to be fully examined. However, this is beyond the scope of this review. Here, we briefly discuss cytokines gaining prominence as critical mediators of airway inflammation in SA, namely IL-33, IL-17, TNF and IFNγ.

IL-33 is a member of the IL-1 family of cytokines, is a potent activator of ILC2s, and primarily promotes steroid-resistant eosinophilic inflammation (6, 22, 26, 42). IL-33 is typically sequestered in the nucleus and released as an “alarmin” following cell injury and stress, as well as in response to allergen exposures. Proteases in environmental allergens can cleave the full length IL-33 to release its mature inflammatory form (55). The cleaved extracellular form of IL-33 engages the ST2 receptor to activate ILC2s, resulting in the induction of eosinophilic inflammation in response to allergens (55, 56). Although, many immune cells such as macrophages, dendritic cells, eosinophils and various subsets of T-cells also express the ST2 receptor (56), the interaction of IL-33 via the ST2 receptor on these immune cell types, and consequent downstream responses are not fully understood. Interestingly, chronic exposure to IL-33 results in a “memory” CD4+ Th2 cell type that preferentially produce IL-5, resulting in eosinophilic airway inflammation (56). We have recently shown that IL-33 challenge in a murine model can induce the production of IL-5 in the lungs (57), which suggests a mechanism induced by IL-33 to augment IL-5 production. Overall, the interplay of IL-33 and IL-5 signaling pathways promote eosinophilic inflammation resistant to ICS therapy in SA (7). In addition, the combinatorial effect of IL-33 with leptin, an obesity-related adipokine, promotes eosinophilic airway inflammation in obesity-related SA (58). Moreover, IL-33 can also stimulate mast cells to enhance Th17-mediated responses in neutrophilic inflammation (59). This is corroborated by various clinical studies, both in children and adults, which correlate the levels of IL-33 with asthma disease severity and steroid-unresponsiveness (23–25, 60, 61). Consequently, IL-33 is defined as a biomarker and a therapeutic target for SA (62, 63).

Another biomarker associated with SA is IL-17. Clinical studies show enhanced levels of IL-17, primarily IL-17A and IL-17A/F, in the lungs, serum and peripheral blood-derived mononuclear cells, of patients with SA (6, 64–67). Moreover, enhanced abundance of IL-17 in the lungs positively correlates with asthma severity, and is not mitigated by steroid treatments (6, 13, 33, 66, 68). IL-17 contributes to the Th2-low/Th17-high immunophenotype characterized by neutrophilic airway inflammation in SA (6, 69). IL-17 produced from Th17 cells recruit neutrophils to the lungs and promote steroid-resistant, neutrophilic airway inflammation (13). Similarly, IL-17 produced by ILC3 cells also leads to a steroid-resistant phenotype, which is associated with obesity-related asthma (70). Thus, both IL-33 (discussed above) and IL-17 have been shown to be critical cytokines associated with promoting airway inflammation in obesity-related SA. Inflammatory processes related to obesity and asthma are thought be the underpinning bridge in obesity-related SA [reviewed in (71)]. Although obesity is associated with SA, immunomodulatory mechanisms of obesity-related SA are not completely defined.

Immunoreactive IL-17 signals through IL-17-receptors expressed on airway structural cells such as bronchial epithelial cells, resulting in the induction of neutrophil chemoattractants which enhances neutrophilic airway inflammation (69). These studies substantiate the critical role of IL-17 in the disease process of SA. Recent studies have shown that Th17-derived IL-17 can induce the expression of the steroid-unresponsive GRβ isoform to promote steroid resistance (66, 67). Although, the role of IL-17 in facilitating SA is now well established, there are no effective therapies that can mitigate steroid-resistance by targeting either IL-17 or associated neutrophil signaling to effectively control SA (72, 73). This also point to the emerging theme that the immune networks involved in the etiology and pathophysiology of the different endotypes of SA are disparate and complex. Previous studies clearly suggest that different cytokine interactions with mixed leukocyte profiles promote various immunophenotypes in SA (66, 74).

Interaction of IL-17 with TNF, a Th1-effector cell cytokine, also drives Th17-inflammation and steroid-resistance, resulting in a mixed Th1/Th17-mediated response in SA (12, 75). TNF is elevated in the sputum, and TNF-receptors (TNFR1 and TNFR2) are enhanced in the sputum and serum of patients with SA, and are generally associated with neutrophilic inflammation (12, 76). The importance of TNF in SA is reinforced by a recent study demonstrating that treatment with azithromycin, which intervenes in TNF dysregulation, suppresses TNF and TNFR2 in SA (76). Aligned with this, a randomized clinical trial also demonstrated that azithromycin can control exacerbations in SA (77). TNF predominantly promotes airway inflammation in SA (12). However, elevated TNF in the lungs can also induce MMP9 production from bronchial epithelial cells, engaging the TNFR1-TRAF2 axis involving protein kinase C and c-Jun / Src kinase signaling pathways (78). As MMP9 promotes fibrosis and decline of lung function in SA (described above), a consequence of elevated TNF may be associated with lung remodeling in SA. Contrary to this, neutralization of TNF does not improve AHR, only improves airway inflammation, in animal model studies (12). Therefore, a direct role of TNF facilitating lung remodeling and AHR via MMP9 production cannot be definitively stated.

Synergy of different cytokines has been defined to augment steroid-resistance in asthma (Figure 1). The synergy of IFNγ with various inflammatory mediators is described in the pathobiology of SA. For example, TNF synergizes with IFNγ to mediate steroid-resistance in asthma (79). Integrated signaling of IFNγ with LPS induces neutrophilic inflammation and macrophage-dependent steroid-insensitive AHR (80). Similarly, IFNγ together with IL-27 mediate steroid-resistance and AHR in SA (81). It is thus not surprising that IFNγ is found to be increased in sputum and blood-derived cells from patients with SA compared to those with mild-to-moderate asthma (64). Similarly, high levels of IFNγ with Th1-high immunophenotype have been demonstrated in the airways of patients with SA (64, 82). Another important biological function of IFNγ, in the context of SA, is its ability to induce the production of IL-33 from airway smooth muscle and bronchial epithelial cells (25, 83). IL-33 is a critical mediator of the steroid-refractory phenotype (discussed above). Therefore, IFNγ may be also contributing to the pathobiology of SA by regulating the production of IL-33 in the lungs.

Overall, studies characterizing the role of different cytokines in SA suggest that interactions of different cytokines lead to complex signaling networks resulting in disparate immunophenotypes in the disease process of SA (Figure 1). Thus, targeting a single cytokine or signaling cascade may not be effective as an intervention strategy for all SA patients. In order to gain insight into the complex signaling networks, several international consortiums have used various omics-based approaches to identify drug targets for SA (84). It is likely that common hubs or nodes within overlapping immune networks associated with the different immunophenotypes may be useful as drug targets for SA. A challenge will be to maintain the beneficial aspects of immune responses while targeting critical nodes within immune networks in order to control SA.

### Molecular Mechanisms of Steroid-Unresponsiveness

Anti-inflammatory effects of glucocorticoids (type of corticosteroids) are mediated by binding to the GR isoform GRα, followed by the translocation of GRα from the cytoplasm to the nucleus to regulate gene transcription (6). Whereas the GRβ isoform located in the nucleus does not bind to steroids, and also attenuates GRα function (85). A primary mechanism of steroid-resistance in SA is by the dysregulation of GR function, which can be mediated by the cytokines involved in the pathobiology of SA (discussed above). For example, a primary mode of action of TNF in SA is to promote increased expression of the GRβ isoform, which changes the GRα/GRβ ratio making GRβ the dominant isoform thus resulting in steroid-resistance (86). Similarly, Th17-derived IL-17 induces the expression GRβ to mediate steroid resistance (66, 67). IFNγ together with IL-27 suppresses the nuclear translocation of GRα in response to glucocorticoids, to induce the steroid-refractory phenotype and AHR in SA (81). These studies clearly demonstrate that specific cytokines that are integrally associated with the various immunophenotypes of SA can facilitate dysregulation of GR-mediated response to steroids (Figure 1).

Another mechanism of anti-inflammatory effects of glucocorticoids is via the induction of a dual phosphatase, protein kinase phosphatase 1 (MKP1), which attenuates pro-inflammatory gene transcription by dephosphorylating p38 MAPK (87). It is important to note that the anti-inflammatory effects of MKP1 is dependent on the kinetics of its mode of action, and post-translational modifications, thus MKP1-mediated functions may not be always anti-inflammatory (88). Nevertheless, the steroid-unresponsiveness phenotype is also thought to be mediated by the impairment of MKP1 function (88, 89). Thus, a higher concentration of steroids is required to induce MKP1 in patients with SA compared to those with steroid-sensitive disease (89). Note that critical elements of innate immunity, NLRP3 inflammasome and IL-1β, also contribute to steroid-resistance, with NLRP3 expression enhanced in SA patients with neutrophilic inflammation (28, 29). TNF, which can mediate steroid-unresponsiveness (86), also regulates the expression of NLRP3 and MKP1 albeit with different kinetics (90). These studies suggest integral links between innate immune responses and regulatory pathways that underpin steroid-unresponsiveness, the immunobiology of which remains to be determined. Recently, epigenetic regulations primarily by microRNAs miR-9, miR-21 and miR-126 have been defined as molecular mechanisms in the process of steroid-resistance (91, 92). Thus, there is emerging interest in examining interventions that modulate microRNA-mediated epigenetic regulation of inflammasome and other immune pathways for the control of SA. It is clear that further studies are needed to unravel regulatory mechanisms and immune networks that control steroid-resistance, and to better understand how these are related to the various immunophenotypes in SA, in order to develop new therapies for effective management of the disease.

## Summary

Fundamental immunobiology of SA is extremely complex and heterogenous, with various immunophenotypes defined from patient cohorts and animal studies. The immune heterogeneity in the disease process is a considerable obstacle in developing new therapeutic approaches to efficiently mitigate SA and/or overcome steroid-resistance. It is clear that a comprehensive understanding of immune networks that contribute to the pathogenesis and regulation of inflammatory phenotypes in SA is critical to gain insight into the biological processes related to heterogeneity in SA. Furthermore, as studies emerge detailing the functions and signaling mechanisms of specific cytokines e.g., IL-17 and IL-33, or other immune mediators, on the initiation, persistence and exacerbation of SA, the utility of these immune effector elements as biomarkers or therapeutic targets will also need to be examined in the context sex and population-associated immunogenetics. It may well be that combination therapies or personalized approaches will be needed for different patient groups with different disease immunophenotypes to effectively control SA. Nevertheless, detailing immune networks in SA is an unmet clinical need, which is critical for the identification of new drug targets and intervention strategies to alleviate the disease process in SA.

## Author Contributions

CM and KH wrote sections of the original draft. NM conceptualized the scope of the review, compiled the original draft, and extensively edited the manuscript. All authors contributed to the article and approved the submitted version.

## Conflict of Interest

The authors declare that the research was conducted in the absence of any commercial or financial relationships that could be construed as a potential conflict of interest.

## Publisher's Note

All claims expressed in this article are solely those of the authors and do not necessarily represent those of their affiliated organizations, or those of the publisher, the editors and the reviewers. Any product that may be evaluated in this article, or claim that may be made by its manufacturer, is not guaranteed or endorsed by the publisher.
